# Detection of Irregular Loads Using SAW Delay-Line Devices

**DOI:** 10.3390/s26072237

**Published:** 2026-04-04

**Authors:** Yining Yin, Zheng Zhao, Ran You, Yong Liang, Wen Wang

**Affiliations:** 1 State Key Laboratory of Acoustics and Marine Information, Institute of Acoustics, Chinese Academy of Sciences, Beijing 100190, China; zhaozheng@mail.ioa.ac.cn (Z.Z.); youran@mail.ioa.ac.cn (R.Y.); liangyong@mail.ioa.ac.cn (Y.L.); 2 University of Chinese Academy of Sciences, Beijing 100049, China

**Keywords:** non-uniform loading, P-matrix array model, propagation characteristic

## Abstract

**Highlights:**

**What are the main findings?**

- The traditional simulation method for the SAW delay-line device response was improved by channelization, enabling analysis of the device response characteristics under different spatial distributions.
- As the load coverage area increases, the phase and frequency responses vary linearly with the load area, while the amplitude-frequency characteristics remain stable and distortion-free.

**What are the implications of the main findings?**

- SAW delay line devices have inherent immunity to distortion under microscale irregular loads and have potential advantages in single-cell-level bioparticle mass detection applications.

**Abstract:**

A two-dimensional segmentation model based on the P-matrix array was developed to simulate surface acoustic wave (SAW) delay-line devices under irregular loading. Building on coupling-of-modes (COM) theory and P-matrix model, a channelization approach was introduced to enhance conventional response simulation, enabling the systematic extraction of frequency and phase characteristics under varying spatial load distributions. Experimental verification was conducted using SAW devices fabricated by depositing aluminum interdigital transducers (IDTs) on Y-cut 35° quartz crystals through semiconductor lithography. The results demonstrate that the two-dimensional segmentation method effectively and accurately simulates the response of SAW delay line devices under various non-uniform and irregular mass loading distributions, both the phase shift and frequency shift exhibit linear proportionality to the loaded area (R^2^ > 0.99), while the amplitude-frequency characteristics remain stable with increasing load coverage, showing no observable distortion or aberration. Quantitative mass detection experiments employing polystyrene microspheres further demonstrate that the device response increases linearly with the number of sample injections, and the shift magnitude is directly proportional to the amount injected per loading event.

## 1. Introduction

In the 1970s, researchers [1] found that surface acoustic waves (SAWs)—mechanical waves propagating along the medium surfaces with energy concentrated within one to two wavelengths from the interface—exhibit extreme sensitivity to external perturbations such as temperature, pressure, magnetic fields, and mass loading. Concurrently, SAW devices emerged as promising sensors, due to their compact size, low power consumption, wireless passive detection capability, and seamless integration with external circuits [2,3,4,5,6,7,8,9]. In 1979, Wohltjen et al. [10,11] pioneered organic gas detection by coating SAW delay-line devices with polymer sensing films, marking the inception of systematic SAW sensing research. For adsorption-based sensors (e.g., gas [8,12,13,14,15], particulate [16,17,18,19], and biosensors [5,20,21,22]), enlarged sensing areas improve both sensitivity and practical utility. Nevertheless, larger sensitive regions in sensor design increase their vulnerability to non-uniform load distribution, which is particularly critical in bioparticle mass detection involving cells, microorganisms, and macromolecules [23].

The spatial distribution of load exertion directly influences SAW device responses. Current SAW device simulations are typically limited to scenarios with fully uniform analyte deposition along the aperture, without accounting for spatial load variations. To enable precise simulation of highly non-uniform loads—such as cells and microorganisms—developing novel SAW device models incorporating two-dimensional spatial load distributions is essential.

L. Solie et al. [24] developed a multi-channel aperture segmentation method for sectorial SAW filters during fabrication, integrating spatial inhomogeneities in interdigital periodicity into frequency response simulations. You et al. [23,25,26] extended this approach by proposing a two-dimensional segmentation method that accounts for spatial load distribution, enabling the analysis of SAW resonator responses under non-uniform loads with experimental validation, and found that when the load exhibits a non-uniform distribution along the acoustic aperture direction, the frequency response curve of the device undergoes waveform distortion accompanied by a splitting of the main peak.

This study employed an arrayed P-matrix model to simulate SAW delay-line device responses under varying spatial load distributions. Results reveal that delay-line devices exhibit superior resistance to waveform distortion under non-uniform loads compared to resonator-type devices, making it more suitable for applications in single-cell-level particle mass detection. To evaluate the simulation framework under complex loading conditions, non-uniform and irregular mass loading distributions were systematically designed and tested, with quantitative particle mass detection experiments being conducted using polystyrene microspheres.

## 2. Theoretical Analysis

**Figure 1:**
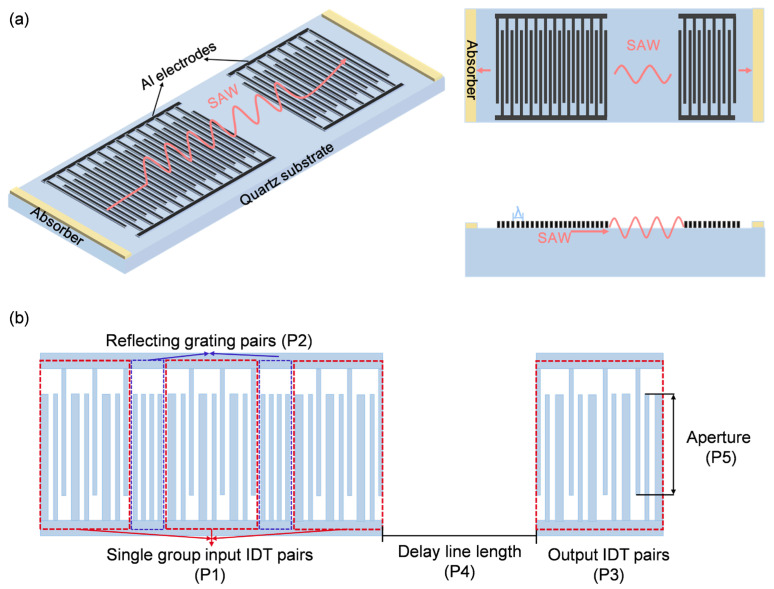
(**a**) Structure and principle of SAW delay-line device. (**b**) Device structure of SAW SPUDT delay line.

SAW devices typically incorporate dedicated sensing regions, with delay-line types employing inter-transducer delay zones and resonator-type configurations utilizing transducer-active areas as sensing domains. For detection applications requiring extensive sensing coverage (e.g., gas, icing, biological, and electromagnetic monitoring), SAW delay-line devices (Figure 1a) are predominantly adopted to accommodate sensitive material deposition and signal acquisition. The delay-line configuration typically consists of two periodic interdigital transducers (IDTs) aligned along the acoustic wave propagation axis, separated by a free surface region for unimpeded wave propagation. Acoustic absorbers are implemented at chip edge terminations to attenuate SAWs reaching the boundaries. The free surface region between IDTs is conventionally designated as the sensing area. Miniaturized analytes exhibiting sub-millimeter dimensions induce non-uniform spatial load distributions on SAW surfaces, necessitating advanced analytical models for characterizing device responses under heterogeneous loading configurations. The SAW delay-line device configuration decomposes into three P-matrices: input IDT, inter-transducer gap, and output IDT. The input IDT incorporates electrode width control (EWC)-optimized single-phase unidirectional transducers (SPUDTs) to achieve low insertion loss, single-mode operation, and linear phase characteristics within the passband, as illustrated in Figure 1b. Apodization processing of the input transducer generates multiple EWC/SPUDT-structured IDT arrays, while the output transducer remains non-apodized. The output transducer length equals the combined length of a single apodized IDT group and reflector grating from the input transducer. Critical design parameters comprise: P1 (finger pairs per apodized IDT group), P2 (reflector grating finger pairs/apodization gap length), P3 (output IDT finger pairs), P4 (delay-line length), and P5 (acoustic aperture).

### 2.1. Coupling of Modes Theory

The coupling-of-modes theory postulates the coexistence of forward-propagating and backward-propagating SAWs along the SAW device’s longitudinal propagation path. Multiple reflections induced by the metallic grating array on the substrate surface create mutual coupling between forward wave R and backward wave S, establishing the fundamental coupling-of-mode equations as follows [27,28]:

(1) $$\left\{\begin{array}{c} \frac{dR}{dx}=-jk_{E}R+j\kappa_{R}S+j\alpha_{R}V\\ \frac{dS}{dx}=jk_{E}S-j\kappa_{S}R-j\alpha_{S}V\\ \frac{dI}{dx}=j\omega CV-j2\alpha_{S}R-j2\alpha_{R}S \end{array}\right.$$

where $\alpha_{R}$ and $\alpha_{S}$ denote forward and backward acoustic wave excitation coefficients of the IDT, respectively; $\kappa_{E}\;$ represents the self-coupling coefficient; $\kappa_{R}$ and $\kappa_{S}$ indicate forward/backward wave reflection coefficients; and C corresponds to static capacitance.

### 2.2. P Matrix Mode

Practical SAW devices can be modeled as cascade configurations comprising fundamental components (input IDT, propagation gap, and output IDT) with intricate series-parallel interactions between acoustic and electrical terminals. The essence of P-matrix modeling lies in characterizing each constituent structure through a 3 × 3 matrix encompassing two acoustic ports and one electrical port. Acoustic port interactions are governed by the upper-left 2 × 2 submatrix, termed the scattering matrix (S-matrix), which defines the wave propagation dynamics. Electrical terminal behavior is encoded in element P_33_, with residual elements quantifying acoustic-electro energy interconversion mechanisms:

(2) $$\left[\begin{array}{c} b_{1}\\ b_{2}\\ i \end{array}\right]=\left[\begin{array}{ccc} P_{11} & P_{12} & P_{13}\\ P_{21} & P_{22} & P_{23}\\ P_{31} & P_{32} & P_{33} \end{array}\right]\left[\begin{array}{c} a_{1}\\ a_{2}\\ u \end{array}\right]$$

The P-matrix elements in the above formulation are derived from solutions of the coupling-of-mode (COM) equations, based on their physical interpretations:

(3) $$\left\{\begin{array}{c} P_{11}=\frac{j\kappa_{S}\sin(DL)}{D\cos(DL)+j\Delta \sin(DL)}\\ P_{12}=\frac{{\left(-1\right)}^{2N}D}{D\cos(DL)+j\Delta \sin(DL)}\\ P_{13}=jL\frac{\sin(DL/2)}{DL/2}\frac{j(\Delta \alpha_{S}+\kappa_{S}\alpha_{R})\sin(DL/2)+\alpha_{S}D\cos(DL/2)}{D\cos(DL)+j\Delta \sin(DL)}\\ P_{22}=\frac{j\kappa_{R}\sin(DL)}{D\cos(DL)+j\Delta \sin(DL)}\\ P_{23}={\left(-1\right)}^{2N}jL\frac{\sin(DL/2)}{DL/2}\frac{j(\Delta \alpha_{R}+\kappa_{R}\alpha_{S})\sin(DL/2)+\alpha_{R}D\cos(DL/2)}{D\cos(DL)+j\Delta \sin(DL)}\\ P_{33}=\frac{-4}{D^{3}}\frac{\left[\left(\Delta^{2}+{\left|\kappa_{R}\right|}^{2}\right){\left|\alpha_{R}\right|}^{2}+2\Delta Re\left(\kappa_{S}\alpha_{R}^{2}\right)\right]\left[1-\cos\left(DL\right)\right]}{D\cos(DL)+j\Delta \sin(DL)}+j\omega LC\\ +j\frac{4}{D^{2}}\frac{\left[\Delta {\left|\alpha_{R}\right|}^{2}+Re\left(\kappa_{S}\alpha_{R}^{2}\right)\right]\sin\left(DL\right)}{Dcos\left(DL\right)+j\Delta \sin\left(DL\right)}-jL\frac{4}{\Delta^{2}-{\left|\kappa_{R}\right|}^{2}}\left[\Delta {\left|\alpha_{R}\right|}^{2}+Re\left(\kappa_{S}\alpha_{R}^{2}\right)\right] \end{array}\right.$$

where L denotes the IDT length and N represents the number of finger pairs. Re denotes the real-part operator. D corresponds to the detuning coefficient, where $D=\sqrt{\Delta^{2}-\left|k^{2}\right|},k=\omega /v-j\gamma$, ∆ is the detuning coefficient, γ is the propagation loss of per unit length, and v represents the phase velocity of SAW propagation.

Due to the symmetry of the three terminal network structure, the P matrix has the following reciprocity:

(4) $$P_{21}=P_{12},P_{31}=-2P_{13},P_{32}=-2P_{23}$$

By constructing P-matrices for fundamental components—input IDTs ($P^{TA}$), delay line gaps $\left(P^{G}\right)$, and output IDTs ($P^{TB}$)—cascading these structural units through network integration principles, the matrix $P^{G}$ is cascaded with $P^{TA}$ to form the $P^{L}$ matrix. Subsequently, $P^{L}$ is combined with the $P^{TB}$, and the admittance matrix Y characterizing electrical port transmission properties of SAW devices is derived as follows:

(5) $$\left(\begin{array}{c} I_{1}\\ I_{2} \end{array}\right)=\left(\begin{array}{cc} Y_{11} & Y_{12}\\ Y_{21} & Y_{22} \end{array}\right)\left(\begin{array}{c} V_{1}\\ V_{2} \end{array}\right)$$

where

(6) $$\left\{\begin{array}{c} Y_{11}=P_{33}^{L}+\frac{P_{11}^{TB}P_{23}^{L}P_{32}^{L}}{1-P_{11}^{TB}P_{22}^{L}}\\ Y_{12}=\frac{P_{13}^{TB}P_{32}^{L}}{1-P_{11}^{TB}P_{22}^{L}}\\ Y_{21}=\frac{P_{31}^{TB}P_{23}^{L}}{1-P_{11}^{TB}P_{22}^{L}}\\ Y_{22}=P_{33}^{TB}+\frac{P_{22}^{L}P_{13}^{TB}P_{31}^{TB}}{1-P_{11}^{TB}P_{22}^{L}} \end{array}\right.$$

The frequency response expressions of S-parameters for SAW devices are ultimately derived through network parameter conversion relationships [29]. Taking the two-port admittance matrix as an exemplar, the S-parameter expressions for SAW devices obtained via network parameter conversion are formulated as follows:

(7) $$\left\{\begin{array}{c} IL=20\log\left|S_{21}\right|\\ S_{21}=\frac{-2Y_{21}\sqrt{Y_{01}Y_{02}}}{\left(Y_{01}+Y_{11}\right)\left(Y_{02}+Y_{22}\right)-Y_{12}Y_{21}} \end{array}\right.$$

where $Y_{01}$ and $Y_{02}\;$ represent the characteristic admittance of signal transmission lines. Given the standardized characteristic impedance of 50 Ω for conventional transmission lines, the characteristic admittance is determined as $Y_{01}=Y_{02}=1/50\left(S\right)$.

### 2.3. Arrayed P-Matrix Models

The conventional P-matrix model characterizes SAW device components as “black boxes” with homogeneous spatial properties, where COM parameters remain spatially invariant. This uniformity inherently limits its capability to analyze SAW device responses under non-uniform loading perturbations. This study introduces a channelization methodology into SAW delay-line device simulations, establishing an arrayed P-matrix model that explicitly accounts for spatial load distribution characteristics.

**Figure 2:**
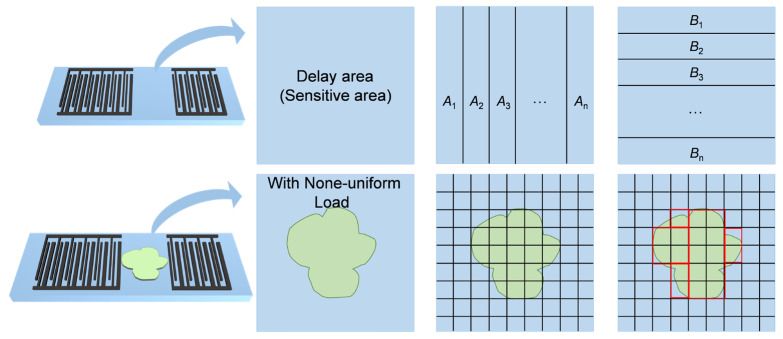
Channelization of sensitive area of SAW delay-line device: according to the actual spatial distribution of the load within the sensing region, the P-matrix of each unit in the array is configured.

Based on the established methodologies, the sensing region of SAW devices is partitioned into the A channel along the acoustic aperture direction, with each channel further subdivided into B along the wave propagation direction, where each unit’s port transmission characteristics are described by a P-matrix. Adjacent P-matrices within channels follow cascade relationships: acoustic ports in series and electrical ports in parallel between IDTs. Ultimately, the SAW device’s sensing region forms a two-dimensional array of P-matrix units, as illustrated in Figure 2. Each unit’s P-matrix elements are independently configurable, enabling computational simulation of SAW delay-line device responses under non-uniform spatial loads through the matrix parameter assignment, which is aligned with actual analyte distribution patterns across the sensing array.

Under channelization processing, SAW can be approximated as planar wave transmission, with weak inter-channel coupling being neglected. This configuration establishes independent acoustic ports and parallel-connected electrical ports across channels, resulting in the admittance matrix Y of the SAW device and the admittance of each channel after channelization $Y_{m}$ satisfying the following:

(8) $$Y=\sum_{n=1}^{m}Y_{m}$$

## 3. Simulation

To ensure accurate extraction of COM parameters, the finite element method (FEM) was employed to compute the boundary frequencies, stopband frequencies, and static capacitance of periodic gratings, followed by a numerical simulation of the COM parameters. The SAW device’s sensing region was ultimately discretized into a two-dimensional array comprising multiple sensing units. Each sensing unit maintains independent COM parameters and corresponding P-matrix configurations. The core concept for the accurate simulation of small-scale complex-distributed loads lies in the flexible configuration of COM parameters across individual sensing units.

A three-dimensional periodic finite element model incorporating paired IDTs was developed using COMSOL Multiphysics ® (version 6.1). Boundary conditions and solving procedures were implemented following methodologies detailed in reference [30,31]. The piezoelectric crystal was Y-cut 35° quartz, and Al was selected as the simulated loading material. The structural parameters of the model are summarized in Table 1. The mode-coupling parameters for device simulation were defined by Equation (9) [30,32]:

(9) $$\left\{\begin{array}{c} f_{0}=\frac{f_{sc+}+f_{sc-}}{2}\\ v=f_{0}\cdot \lambda =\frac{f_{sc+}+f_{sc-}}{2}\cdot \lambda \\ \left|\kappa_{n}\right|\lambda =2\pi \frac{f_{sc+}-f_{sc-}}{f_{sc+}+f_{sc-}}\\ \left|\alpha_{n}\right|=\sqrt{\omega C_{n}\lambda \pi \left(\frac{f_{sc+}-f_{sc-}}{f_{sc+}+f_{sc-}}-1\right)}\\ \cos(∠\alpha_{n}^{2}/\kappa )=\frac{(f_{oc+}-f_{oc-}{)}^{2}-(f_{sc+}-f_{sc-}{)}^{2}}{2\left(f_{sc+}-f_{sc-}\right)\left[\left(f_{oc+}+f_{oc-}\right)-\left(f_{sc+}+f_{sc-}\right)\right]}\\ -\frac{[(f_{oc+}+f_{oc-})-(f_{sc+}+f_{sc-}){]}^{2}}{2\left(f_{sc+}-f_{sc-}\right)\left[\left(f_{oc+}+f_{oc-}\right)-\left(f_{sc+}+f_{sc-}\right)\right]} \end{array}\right.$$

here, v,$\kappa_{n}$,$\alpha_{n}$ and $C_{n}$ represent propagation velocity, coupling coefficient, excitation coefficient, and static capacitance, respectively. $f_{sc+}$, $f_{sc-}$, $f_{oc+}$ and $f_{oc-}$ denote the upper/lower stopband boundary frequencies for periodic shorted and open gratings. w and λ correspond to acoustic aperture and associated wavelength.

In Table 1, the periodic parameter specifies the periodic length of the IDT structure. The aperture parameter defines the model width, which can be minimized to reduce computational demands. Both the IDT electrode thickness and Al loading thickness in the sensing region measure 5000 angstroms (Å).

**Figure 3:**
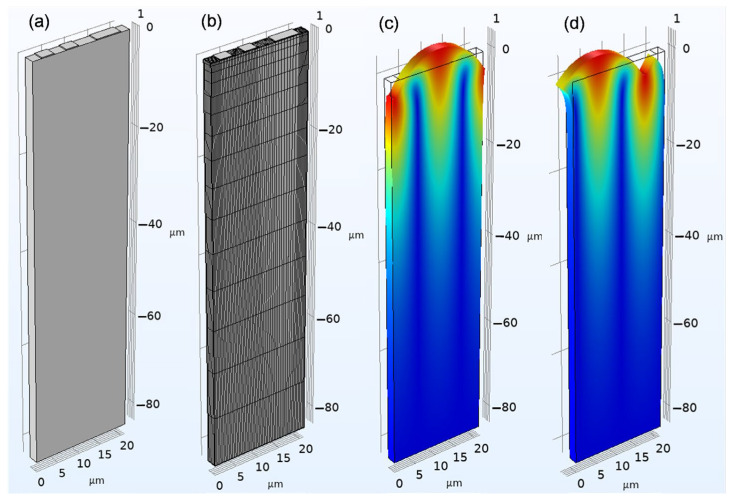
SAW finite element analysis: (**a**) aluminum electrode/Y cut 35° quartz structure. (**b**) Meshing. (**c**) Symmetric and (**d**) antisymmetric modes.

A three-dimensional periodic model of the SAW device was established in the finite element simulation platform COMSOL, as illustrated in Figure 3a. The model comprises a piezoelectric substrate and metallic electrodes. Since the energy of the SAW is primarily concentrated within a depth of 1–2λ from the surface of the piezoelectric substrate, the thickness of the piezoelectric substrate in the model was set to 4λ to reduce the modeling costs and computational burden. Given that the energy of the SAW is primarily concentrated within a depth of 1–2λ from the substrate surface, the thickness of the piezoelectric substrate in the model was set to 4λ to reduce the modeling costs and computational effort. By imposing periodic boundary conditions along both the acoustic propagation direction and the acoustic aperture direction, the infinitely long grating array model was simplified to a single-period model containing only one pair of IDTs. In terms of mesh generation, since the geometric model of the periodic grating array consists of regular rectangles, a mapped-swept method was employed to construct a mesh composed of hexahedral elements. Meanwhile, considering the characteristic that the SAW amplitude decays progressively with the substrate depth, a depth-dependent mesh density distribution was adopted along the substrate depth direction, resulting in a finer mesh near the surface to enhance the solution accuracy (Figure 3b). Symmetric and antisymmetric Rayleigh SAW modes are demonstrated in Figure 3c,d, respectively. The corresponding resonant frequencies (shorted/open grating configurations) determine the stopband boundary frequencies. The COM parameters for loaded IDTs were calculated using Equation (9). Through the above finite element analysis, com parameters under no-load and load conditions were obtained, as shown in Table 2.

**Figure 4:**
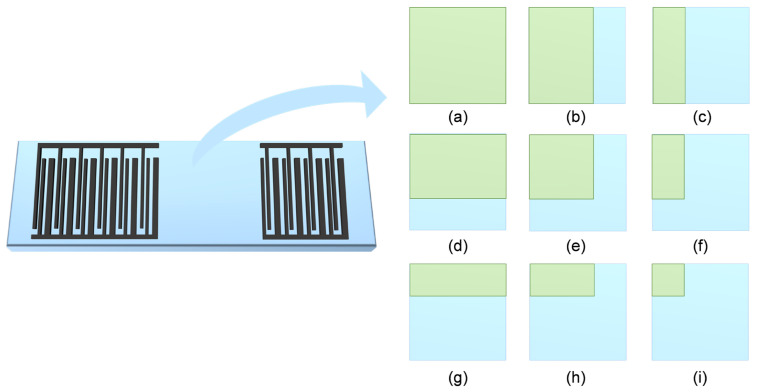
Different load conditions: (**a**) full coverage, 1/3 bidirectional coverage; (**b**) 2/3 transverse and full longitudinal coverage; (**c**) 1/3 transverse and full longitudinal coverage; (**d**) 2/3 longitudinal and full transverse coverage; (**e**) 2/3 bidirectional coverage; (**f**) 1/3 transverse and 2/3 longitudinal coverage; (**g**) 1/3 longitudinal and full transverse coverage; (**h**) 2/3 transverse and 1/3 longitudinal coverage; and (**i**) 1/3 bidirectional coverage.

Building upon the established arrayed P-matrix model, numerical simulations were performed to analyze the frequency responses of SAW devices under complex loading conditions with diverse spatial distribution characteristics. Nine distinct loading configurations were systematically designed (Figure 4), specifically: full coverage over delay-line sensing region (Figure 4a); 2/3 transverse coverage with full longitudinal coverage (Figure 4b); 1/3 transverse coverage with full longitudinal coverage (Figure 4c); full transverse coverage with 2/3 longitudinal coverage (Figure 4d); 2/3 coverage in both transverse and longitudinal dimensions (Figure 4e); 1/3 transverse and 2/3 longitudinal coverage (Figure 4f); full transverse coverage with 1/3 longitudinal coverage (Figure 4g); 2/3 transverse and 1/3 longitudinal coverage (Figure 4h); and 1/3 coverage in both dimensions (Figure 4i). Insertion loss S_21_ parameters for all nine loading patterns were computationally derived, with corresponding frequency response curves being graphically represented.

**Figure 5:**
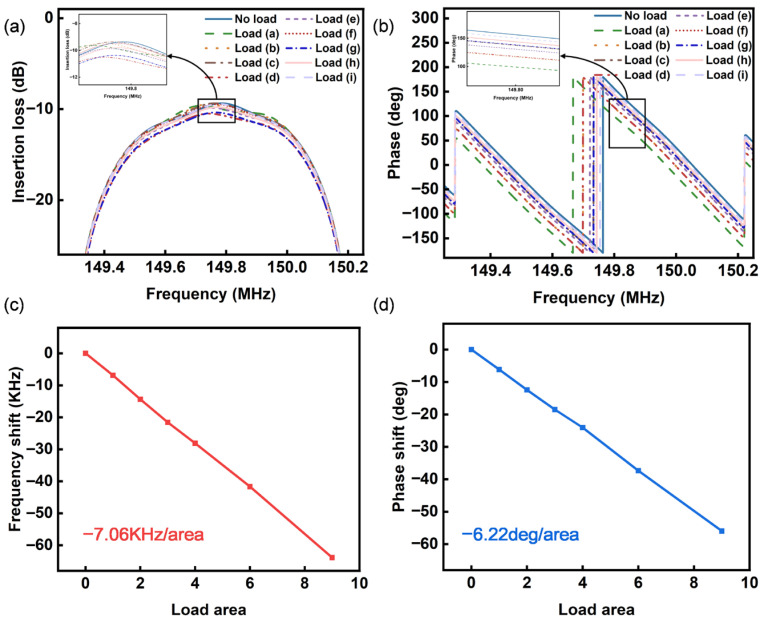
Simulation of frequency offset and phase offset under different load conditions: (**a**) frequency response. (**b**) Phase response. (**c**) Frequency offset. (**d**) Phase offset.

For loading pattern-a (full sensing area coverage), the primary resonance peak shifts leftward with a 63.8 kHz center frequency reduction. Loading patterns-b/d (2/3 longitudinal/transverse coverage) demonstrate 42.0 kHz center frequency reductions with analogous leftward peak shifts. Patterns-c/g (1/3 coverage) exhibit 21.6 kHz and 20.8 KHz frequency decreases and consistent leftward peak migration. Pattern-e (4/9 coverage) shows 28.1 kHz center frequency attenuation. Patterns-f/h (2/9 coverage) display 14.3 kHz and 13.7 kHz frequency shifts. Pattern-i (1/9 coverage) reveals 6.9 kHz frequency deviation. Quantitative analysis confirms the linear proportionality between the loading area and frequency shift magnitude. The frequency sensitivity is −7.06 KHz/area. All loading configurations maintain waveform integrity without spectral distortion during center frequency migration. This stability originates from SAW delay-line devices’ inherent linear phase regime. A phase detection methodology is conventionally employed for response signal acquisition in sensing applications. Figure 5b illustrates the phase-frequency response curves, demonstrating preserved linear phase characteristics across all nine loading patterns. The measured phase shift values at 149.8 MHz are as follows: 55.9 deg, 37.3 deg, 18.3 deg, 37.3 deg, 24.1 deg, 12.5 deg, 18.7 deg, 12.2 deg, and 6.2 deg, respectively. The frequency sensitivity is –6.22 deg/area.

## 4. Experiments and Discussion

### 4.1. Non-Uniform Load Experiment

According to the theoretical analysis model and numerical calculation results from the response of SAW delay-line devices under non-uniform distributed loads established earlier, it can be concluded that non-uniform mass loading induces proportional frequency/phase shifts in SAW delay-line devices while preserving waveform integrity without spectral distortion. To verify the accuracy of the theoretical analysis mentioned above, this section conducted experimental research on the frequency response of SAW devices under loads with different spatial distribution patterns.

**Figure 6:**
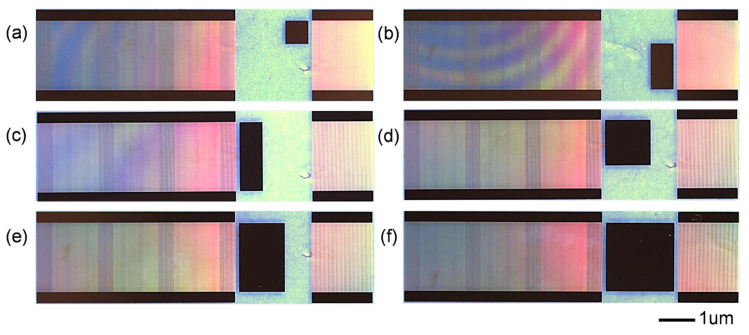
Images taken by SAW delay-line microscope under six load modes: (**a**) 1/3 bidirectional coverage; (**b**) 1/3 transverse and 2/3 longitudinal coverage; (**c**) 1/3 transverse and full longitudinal coverage; (**d**) 2/3 bidirectional coverage; (**e**) 2/3 transverse and full longitudinal coverage; and (**f**) full coverage.

Six representative loading patterns were fabricated using semiconductor lithography to deposit 5000 Å aluminum (Al) layers on designated SAW delay-line regions, followed by pre/post-loading S_21_ parameter characterization across 149–151 MHz; the microscopy images of SAW delay-line under various loading patterns are presented in Figure 6, with black regions indicating the Al loading layers.

**Figure 7:**
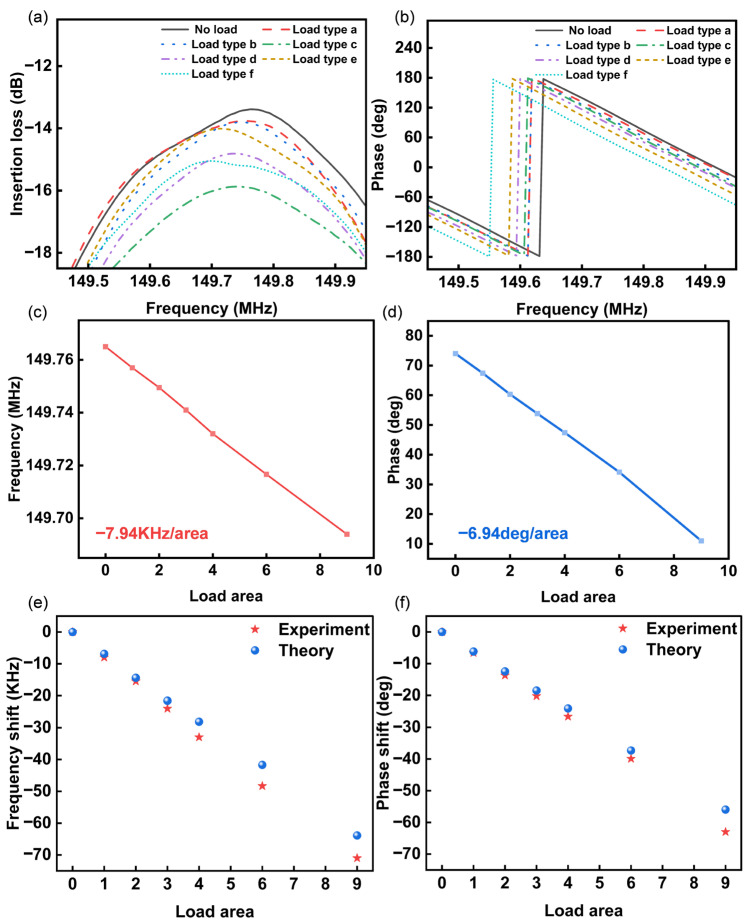
Measured results of SAW delay-line frequency response curves under six load modes: (**a**) frequency response. (**b**) Phase response. (**c**) Frequency offset. (**d**) Phase offset. (**e**) Frequency and (**f**) phase comparison between experiment and theoretical calculation.

Frequency and phase response curves under varied loading conditions were characterized using the vector network analyzer (Keysight E5061B, Santa Clara, California, USA), as shown in Figure 7a,b. The results reveal a preserved main lobe morphology with leftward shifted center frequencies: 71.2 kHz, 48.3 kHz, 33.1 kHz, 24.0 kHz, 15.6 kHz, and 8.0 kHz reductions, relative to unloaded states. Phase shifts at the fixed 149.8 MHz measurement point are 63.0 deg, 39.9 deg, 26.6 deg, 20.2 deg, 13.6 deg, and 6.6 deg, respectively. The calibrated sensitivities (Figure 7c,d) were −7.94 KHz per unit area (frequency) and −6.94 deg per unit area (phase). The experimental data exhibits excellent agreement with the simulation results in Figure 5c,d, as shown in Figure 7e,f, exhibiting ~12% deviation from theoretical predictions. This discrepancy primarily originates from fabrication tolerances and measurement uncertainties.

**Figure 8:**
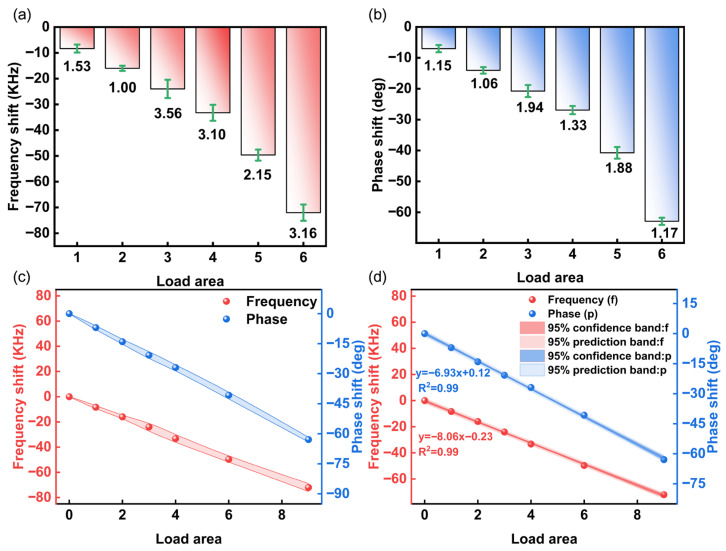
(**a**) Frequency offset and deviation. (**b**) Phase offset and deviation at different positions under the same load. (**c**) Error bands of frequency and phase. (**d**) The 95% confidence and prediction bands for frequency and phase.

Repeatability tests were subsequently conducted for devices at varied spatial positions under identical loading conditions. Group testing covered Loading Patterns 1–6 (mass ratios 1:2:3:4:6:9) across different locations under consistent loading magnitudes. For each case, depending on the device yield, three to five replicate measurements were conducted. The statistical analysis results are presented in Figure 8a–d. The maximum standard deviation of frequency measurements was 3.56 kHz across spatial positions. Phase detection exhibited 1.94 deg maximum standard deviation. This demonstrates that SAW delay-line detection depends solely on loading magnitude, independent of spatial location within the sensing region. Response magnitudes scale linearly with the applied load, with excellent linear regression goodness (R^2^ > 0.99). The primary sources of experimental measurement error in this work are twofold. Firstly, there is an error inherent in the measurement readings themselves, as the recorded values represent discrete instantaneous snapshots at specific moments in time. Secondly, variability was introduced during the device fabrication process. At a 95% confidence level, the frequency sensitivity was determined to be −8.06 KHz/area, and the phase sensitivity was −6.93 deg/area.

### 4.2. Irregular Load Experiment

**Figure 9:**
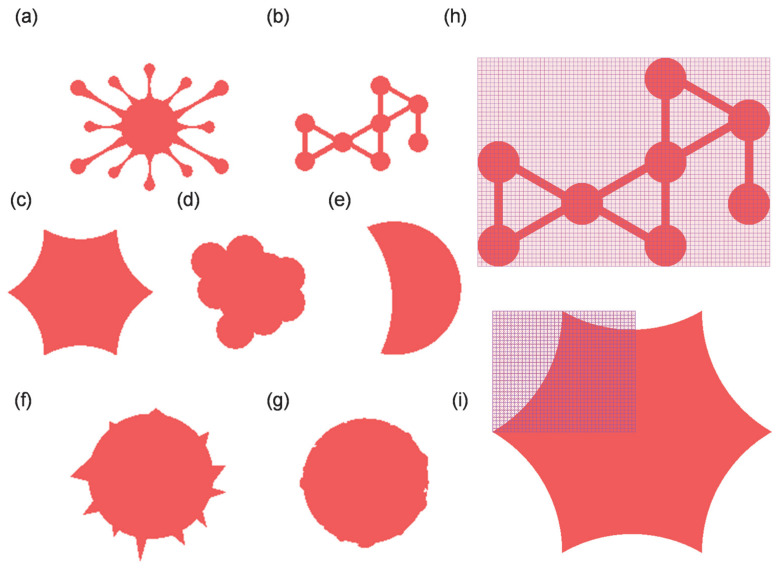
Multiple irregular load shapes simulating cellular distributions (**a**–**g**) and examples of mesh partitioning (**h**,**i**).

To further validate the practical performance of the two-dimensional coupling-of-modes (COM) model for SAW delay lines with non-uniform loading, seven distinct irregular load shapes were designed as depicted in Figure 9a–g. Silicon dioxide (SiO_2_) was employed as the simulated load material. Initially, the COM parameters for the SiO_2_-loaded condition were extracted. Subsequently, the aforementioned model was utilized to perform meshing on the loads (Figure 9h,i); the count of grid cells completely filled by the load is directly recorded; and for the remaining partially filled cells, the grid-filling algorithm (‘complementary 1’ method) is applied: partially filled cells occupying over half a grid cell area are combined with those occupying less than half a grid cell area, and the pair is counted as one full cell. Any remaining isolated cell occupying less than half a grid cell area is discarded; alternatively, any isolated cell occupying over half a grid cell area is counted as one full cell. Load regions exceeding and falling below half the area were integrated accordingly. Frequency and phase shifts under each load condition were then calculated by combining the P-matrix cascading method. The corresponding devices were fabricated, and the SAW device responses were measured using the network analyzer.

**Figure 10:**
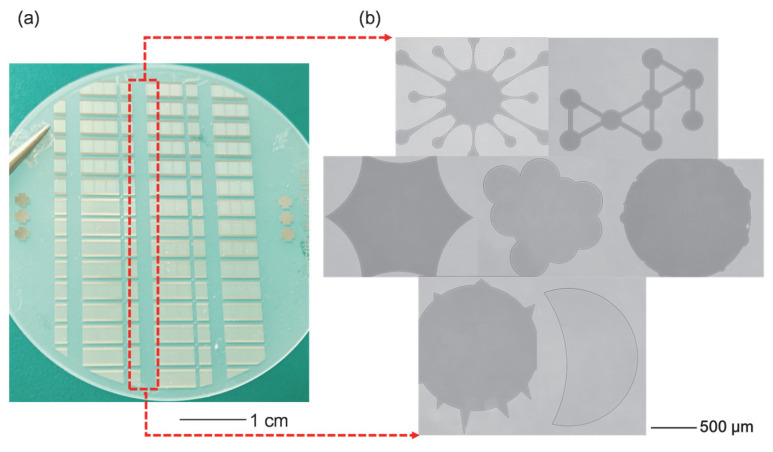
(**a**) Wafer diagram. (**b**) Micrographs of irregular non-uniform load patterns.

The wafer map of the fabricated devices with irregular non-uniform loads is presented in Figure 10a. Micrographs captured by a microscope (LEICA DCM8, Germany) showing different load patterns within the delay-line region are shown in Figure 10b.

**Figure 11:**
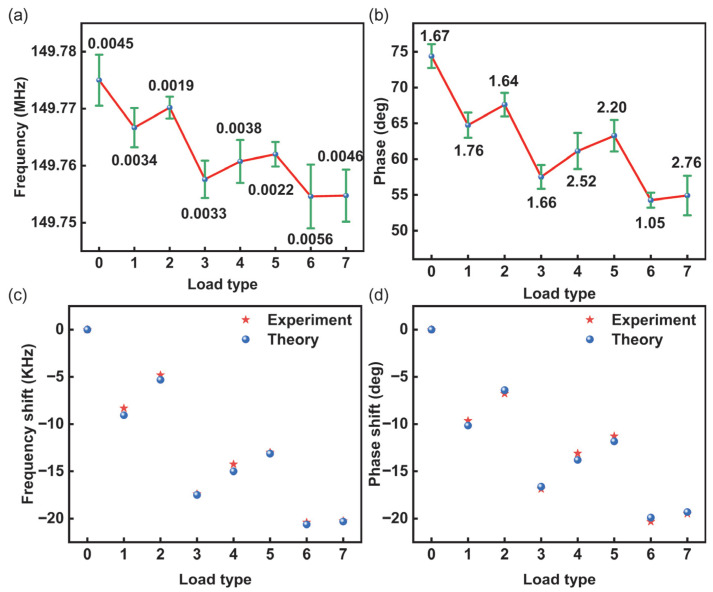
Multiple-load experimental results: (**a**) frequency. (**b**) Phase. (**c**) Frequency and (**d**) phase comparison between experiment and theoretical calculation.

For each shape, SAW device testing was repeated more than three times to ensure the reliability of the experimental results. The relationship between each load shape and the SAW device’s frequency and phase is recorded in Figure 11a,b, in which the mean experimental responses and associated error bars are plotted. The frequency error was less than 5.60 kHz, and the phase error was less than 2.76 deg. The results indicate that the response under different loading conditions is proportional to the load area. A further comparison between the experimental results and the theoretical model predictions is presented in Figure 11c,d. The experimental results demonstrate good agreement between theory and experiment for the SAW delay-line sensing elements under these loading conditions, with frequency errors being less than 8% and phase errors less than 5%. This validates the feasibility of the two-dimensional COM model for sensing non-uniform loads.

### 4.3. Particle Quality Experiment

**Figure 12:**
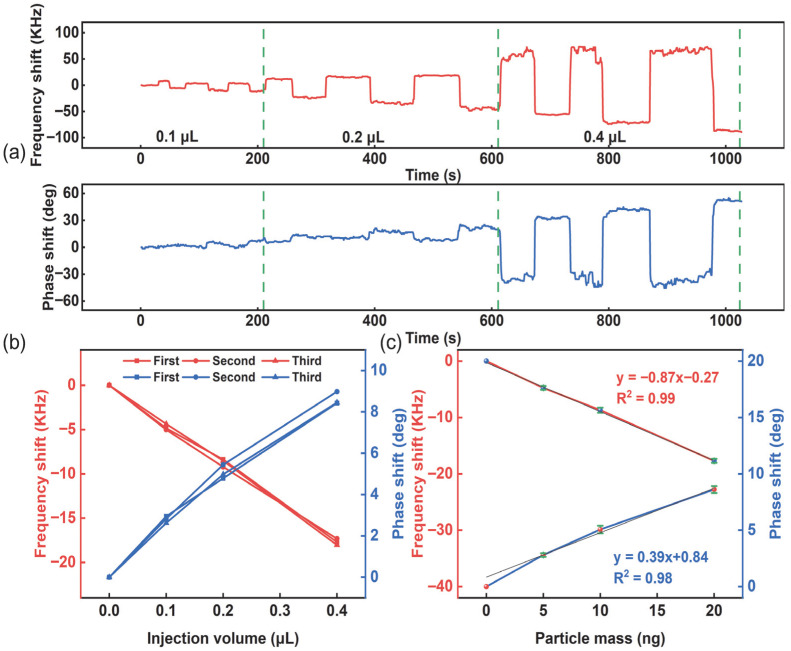
Particle quality experimental results: (**a**) real time responses of frequency and phase. (**b**) Frequency shift and phase shift in the SAW device for each injection event. (**c**) The relationship between the frequency and phase response values of the SAW device and the mass of particles.

Quantitative experiments targeting particle mass detection were conducted, with each test involving repeated quantitative injections at non-fixed positions. Polystyrene microspheres were employed as the experimental subject. A 0.1 mL volume of a 5 mg/mL polystyrene microsphere solution was transferred into 10 mL of distilled water for dilution, yielding a final solution with a mass concentration of 50 ng/µL. The prepared solution was thoroughly mixed using a vortex mixer, and aliquots of 0.1 µL, 0.2 µL, and 0.4 µL were dispensed onto the delay line region of the SAW sensor using a micropipette, corresponding to injection masses of 5 ng, 10 ng, and 20 ng, respectively. Each injection mass was cumulatively administered three times. A vector network analyzer (E5061B, Keysight) with standard commands for programmable instruments (SCPI) control protocol was employed to detect and record variations in the propagation loss of the SAW device, as shown in Figure 12a. After complete solvent evaporation, the corresponding frequency shift and phase shift in the SAW device following each injection were recorded (Figure 12b). The results in Figure 12c demonstrate that the EWC/SPUDT-type SAW delay line sensing element used in this study achieved a frequency-mass sensitivity of 0.87 kHz/ng and a phase-mass sensitivity of 0.38 deg/ng in the particle mass detection application.

### 4.4. Discussion of Limitations

The two-dimensional P-matrix segmentation model simulates the overall system behavior by cascading individual element matrices. This approach significantly reduces the computational complexity associated with traditional full-wave simulations and enables the modeling of non-uniform mass loading.

In the proposed 2D segmentation model for non-uniformly distributed loads, the propagating SAWs are treated as plane waves; consequently, neglecting the weak inter-channel coupling and assuming independent acoustic ports with parallel electrical connections leads to an underestimation of insertion loss and an inability to predict parasitic responses. Additionally, in practical applications, applying a non-uniform load on the IDTs, or when employing a SAW resonator as a sensing device, induces lateral wave velocity variations. This leads to parasitic coupling between adjacent channels. Moreover, when SAW devices are used for liquid-phase detection or aerosol sensing, the viscoelastic load on the sensitive area further exacerbates channel coupling, and the specific biomolecules to the surface forms localized “mass-spring” systems; these systems alter the surface boundary conditions and simultaneously induce acoustic field perturbations in adjacent channels. The 2D P-matrix model struggles to describe this three-dimensional spatial coupling, as it primarily considers energy transfer along the propagation direction, neglecting energy penetration in the surface normal direction and lateral acoustic leakage. A multiphysics coupling model can be established by combining a two-dimensional coupling-of-modes theory (2D COM) with the finite element method, while a process variation statistical model is introduced to compensate for spatial variations in acoustic velocity caused by lithography deviations, material non-uniformity, and electrode thickness fluctuations, thereby improving the model prediction accuracy.

## 5. Conclusions

This study proposes a two-dimensional arrayed P-matrix method for analyzing SAW delay-line devices under non-uniform loading conditions. The validity of the method was confirmed through simulations and experiments under both regular and irregular load distributions. The results demonstrate that this method accurately simulates device responses across various mass-loading patterns, with the phase shift being linearly correlated with the load area and the amplitude-frequency characteristics remaining stable and distortion-free. These findings suggest that SAW devices possess an inherent distortion-resistant capability under microscale non-uniform loading, highlighting their potential for biological mass detection. Future work will focus on integrating the proposed method into practical biosensing applications, along with optimizing sensitivity and resolution for single-cell-level mass detection.

## Figures and Tables

**Table 1:** COM model parameters.

Parameters	Value
Period	21 (μm)
Aperture	1890 (μm)
IDT thickness	5000 (Å)

**Table 2:** COM parameters.

COM Parameters	No Load	Load
Grid array wave velocity (m/s)	3150.84	3150.84
Free surface velocity (m/s)	3153.78	3148.32
Normalized excitation coefficient (Ω^−1/2^)	2.34 × 10^−5^	2.34 × 10^−5^
Normalized Static Capacitance (F/m)	4.50 × 10^−11^	4.50 × 10^−11^
Normalized Coupling Coefficient	4.19 × 10^−4^	4.19 × 10^−4^

## Data Availability

The original contributions presented in this study are included in the article. Further inquiries can be directed to the corresponding author.

## Abbreviations

The following abbreviations are used in this manuscript:

SAW	Surface acoustic wave
COM	Coupling-of-modes
IDT	Interdigital transducer
